# *Staphylococcus aureus* rewires arginine metabolism to drive mammary aging via macrophage–epithelial crosstalk

**DOI:** 10.1371/journal.ppat.1014403

**Published:** 2026-07-06

**Authors:** Yuhang Jin, Kai Yang, Ruoxi Sun, Yu Cao, Dewei He, Junlong Bi, Wenjin Guo, Shoupeng Fu

**Affiliations:** 1 State Key Laboratory for Diagnosis and Treatment of Severe Zoonotic Infectious Diseases, Key Laboratory for Zoonosis Research of the Ministry of Education, Institute of Zoonosis, and College of Veterinary Medicine, Jilin University, Changchun, China; 2 College of Animal Science, Jilin University, Changchun, China; 3 Yunnan Province International Joint Research and Development Center for Veterinary Pharmaceuticals, Yunnan Agricultural University, Kunming, Yunnan, China; INEM: Institut Necker-Enfants Malades, FRANCE

## Abstract

As a common opportunistic pathogen, *Staphylococcus aureus* (*S. aureus*) can rapidly adapt to the host immune system and cause chronic infections. Currently, such chronic infections are extremely difficult to eliminate, severely impairing the function of tissues and organs. We observed localized tissue senescence in the mammary glands of mice with chronic *S. aureus* infection; however, the mechanism driving this senescence remains unclear. To address this, we employed a mouse model of chronic *S. aureus*-induced mastitis and an *in vitro* model of mouse mammary epithelial cells (mMECs) to confirm the inductive effect of *S. aureus* on tissue and cellular senescence. Through integrated analysis of the transcriptome and metabolome of mouse mammary tissues, it was found that the ornithine cycle was significantly disordered following *S. aureus* infection, wherein Arginase 1 (Arg1) serves as a key metabolic regulator. Notably, stimulation by *S. aureus* induces mammary epithelial cells to produce cytokines, thereby promoting the massive release of Arg1 by macrophages into the microenvironment, which constitutes one of the sources of abnormally elevated Arg1 in mammary tissue. This extracellular Arg1 is subsequently taken up by mammary epithelial cells, further accelerating the intracellular conversion of arginine to ornithine. The accumulation of ornithine and its downstream metabolites drives the occurrence of senescence in epithelial cells. Importantly, supplementation with excess arginine or ornithine can mimic this senescence phenotype, while knocking down Arg1 in epithelial cells can reverse this phenomenon. In summary, this study reveals a novel immunometabolic cross-regulation mechanism, specifically that Arg1 released by macrophages—triggered by mammary epithelial cells under *S. aureus* stimulation—acts as a paracrine senescence-inducing factor that acts back on mammary epithelial cells. The Arg1—ornithine axis holds promise as a potential therapeutic target for chronic infection-associated tissue senescence.

## Author summary

This study found that *S. aureus* stimulation induces senescence in mouse mammary glands and mMECs. In mMECs, *S. aureus* directly induces senescence-associated changes, including activation of p53/p21, DNA damage, and SASP-related inflammatory responses, despite reduced endogenous Arg1 expression. Meanwhile, the compensatory upregulation of Arg2 and other ornithine-cycle enzymes suggests that arginine/ornithine metabolism is remodeled in epithelial cells. In the tissue microenvironment, epithelial-derived inflammatory mediators further stimulate macrophages to express and release Arg1. This macrophage-derived Arg1 can be taken up by mMECs and may amplify epithelial senescence by promoting arginine-to-ornithine metabolic dysregulation. Therefore, macrophage-derived Arg1 promotes epithelial senescence but is not the sole initiator of this process.

## Introduction

Senescence is a highly conserved biological process during the evolution of organisms. It is characterized not only by the progressive decline in global physiological functions accumulated over time but also by a direct association with the occurrence and progression of major diseases such as cardiovascular diseases [1], neurodegenerative diseases [2], and malignant tumors [3]. In recent years, in addition to time-dependent senescence, localized senescence of tissues and organs driven by inducers including inflammatory stress and tissue damage has gradually become a research focus in the field of senescence. Chronic inflammation is widely recognized as a core endogenous regulatory factor of localized senescence [4]. Senescent cells secrete the senescence-associated secretory phenotype (SASP)—comprising proinflammatory cytokines, chemokines, and matrix metalloproteinases. These further induce surrounding normal cells to enter a senescent state, triggering a cascading amplification effect [5]. Studies have confirmed that senescent livers can transmit senescent phenotypes to distant organs such as the lungs and kidneys via the TGF-β signaling pathway [6], revealing a key mechanism underlying the spread of localized senescence to systemic effects. *S. aureus*, a ubiquitous opportunistic pathogen in nature, exhibits a colonization rate of approximately 30% in the human population. When the host’s immune barrier is impaired or immune function is compromised, colonizing *S. aureus* can breach immune defenses and cause secondary infections. It is the primary pathogen responsible for clinical conditions such as infective endocarditis, skin and soft tissue infections, pulmonary infections, and implantable medical device-related infections [7]. This bacterium possesses robust environmental adaptability: on one hand, it can enhance drug resistance through mechanisms such as biofilm formation [8]; On the other hand, it can secrete a variety of virulence factors to inhibit the host’s immune response [9]. Meanwhile, *S. aureus* can rapidly adapt to antibiotic selection pressure and continuously acquire antibiotic resistance genes [10]. These characteristics predispose *S. aureus* infections to progress to chronicity [11], the persistent bacterial stimulation over the long term maintains the host tissue in a state of chronic inflammation, which may further induce localized tissue senescence.

In the field of mammary gland diseases, *S. aureus* is generally recognized as one of the primary pathogens of infectious mastitis [12,13]. Clinical studies have shown that patients with chronic *S. aureus*-induced mastitis frequently present with symptoms such as impaired mammary secretory function and disorganized mammary gland structure, suggesting that the functional decline of mammary tissue may be associated with infection. As the functional core of mammary tissue, mammary epithelial cells directly determine the health status of the mammary gland by maintaining their proliferative, differentiative capacities and physiological functions [14,15]. Additionally, the crosstalk between immune cells (macrophages in particular) and epithelial cells within the mammary microenvironment plays a key role in regulating epithelial cell proliferation and cell fate [16]. However, during the process of mammary epithelial cell senescence induced by chronic *S. aureus* infection, how the immune microenvironment participates in the regulatory process, as well as the underlying key molecular mechanisms and signaling pathways, remain unclear. Elucidating this scientific question holds great significance for understanding the pathogenesis of chronic infection-related mammary senescence and developing targeted intervention strategies.

Within the complex tissue microenvironment, macrophages are key immune effector cells that regulate tissue repair and immune responses through the release of cytokines and functional proteins [17,18]. Macrophage arginine metabolic shunting acts as a core molecular switch governing immune phenotype and functional polarization, as well as a critical hub linking immune responses to cellular energy metabolism. Arg1 and inducible nitric oxide synthase (iNOS) are the pivotal enzymes regulating this metabolic bifurcation; they competitively bind the substrate L-arginine and mediate two functionally distinct metabolic pathways [19–21]. iNOS catalyzes arginine to produce nitric oxide (NO) [22], exerting pro-inflammatory and bactericidal effects, Arg1 is a critical metabolic enzyme of the ornithine cycle that catalyzes the hydrolysis of L-arginine into L-ornithine and urea [23–25]. While Arg1 is classically characterized as a cytosolic enzyme [26], recent studies have demonstrated that it can be released into the extracellular space/microenvironment during inflammation or cell death, or via extracellular vesicles (EVs) [27,28]. Arg1 modulates immune function by competitively regulating the metabolic flux of arginine, thereby affecting adjacent cells [29]. Given that Arg1 expression is significantly upregulated in aged tissues [30] and that arginine metabolism is closely associated with immune regulation and cellular homeostasis [31], we hypothesize that metabolic imbalance mediated by aberrant Arg1 expression and dysfunction of the ornithine cycle may represent a key molecular link connecting chronic infection and tissue senescence. This study conducted an in-depth investigation into this phenomenon to explore the underlying regulatory mechanisms.

In this study, we first confirmed that *S. aureus* infection induces senescence and significantly upregulates Arg1 in mouse mammary gland tissues. However, a critical discrepancy emerged in our mechanistic investigation: while Arg1 was highly elevated in infected tissues, *S. aureus* stimulation in vitro failed to upregulate Arg1 expression in mMECs. Transcriptomics and targeted metabolomics focusing on energy metabolism further identified disruption of the ornithine cycle and aberrant upregulation of Arg1. This discrepancy suggests that the elevated Arg1 observed in infected mammary tissue may originate, at least in part, from non-epithelial cells within the mammary microenvironment. Using conditioned-medium co-culture assays, we identified macrophages as an important potential source of extracellular Arg1 under *S. aureus*-stimulated epithelial–macrophage crosstalk. We found that *S. aureus* infection promotes Arg1 release from macrophages, which is subsequently taken up by epithelial cells, leading to metabolic dysregulation of the ornithine cycle and ultimately driving senescence. This study elucidates the molecular mechanisms underlying *S. aureus*-induced mammary senescence, providing a theoretical basis for developing interventions to maintain mammary health and delay mammary senescence.

## Results

### *S. aureus* induces senescence in mMECs

**Fig 1:**
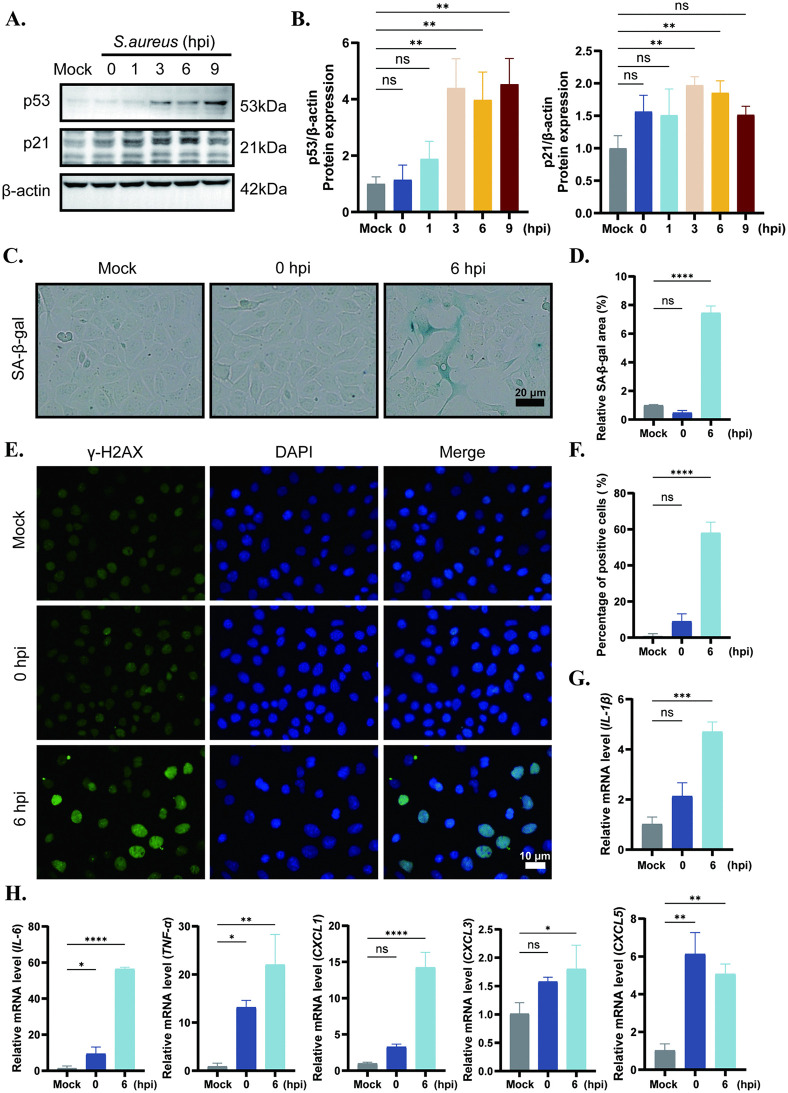
*S. aureus* induces senescence of mMECs. **A.** Western blot analysis of p53 and p21 protein expression in mMECs; **B.** Relative protein levels of p53 and p21 normalized to β-actin; **C.** SA-β-gal staining of mMECs; **D.** Proportion of SA-β-gal-positive area in the staining; **E.** γ-H2AX immunofluorescence staining of mMECs; **F.** Proportion of γ-H2AX-positive cells in the immunofluorescence staining; G-H. mRNA expression levels of *IL-1β*, *IL-6*, *TNF-α*, *CXCL1*, *CXCL3*, and *CXCL5* in mMECs; Data are presented as mean ± standard deviation (n=3). *p < 0.05, **p < 0.01, ***p < 0.001, ****p < 0.0001. ns indicates no significant difference.

First, we stimulated mMECs with *S. aureus* for different durations. Western blot results demonstrated that with the extension of stimulation time, the expression levels of senescence-associated proteins p53 and p21 were significantly upregulated (Fig 1A and 1B). Notably, at 6 h, the expression levels of both proteins were significantly upregulated, with signal intensities far higher than those at the early time points (0 h/1 h). Meanwhile, this time point also avoids the problem of p21 expression decline observed at 9 h, a 6-hour stimulation duration was selected for subsequent experiments. Senescence-associated β-galactosidase (SA-β-gal) staining results revealed that 6-hour stimulation with *S. aureus* increased SA-β-gal-positive staining in mMECs (Fig 1C and 1D). Immunofluorescence assays further showed increased nuclear γ-H2AX signal in S. aureus-stimulated cells (Fig 1E and 1F). Quantitative real-time polymerase chain reaction (qRT-PCR) was performed to detect the senescence-associated secretory phenotype (SASP). The results indicated that *S. aureus* stimulation significantly upregulated the mRNA levels of interleukin-1β (*IL-1β*), interleukin-6 (*IL-6*), tumor necrosis factor-α (*TNF-α*), C-X-C motif chemokine ligand 1 (*CXCL1*), C-X-C motif chemokine ligand 3 (*CXCL3*), and C-X-C motif chemokine ligand 5 (*CXCL5*) in mMECs (Fig 1G and 1H). Collectively, these results suggest that *S. aureus* stimulation induces senescence in mMECs.

### *S. aureus* induces senescence in mouse mammary glands

**Fig 2:**
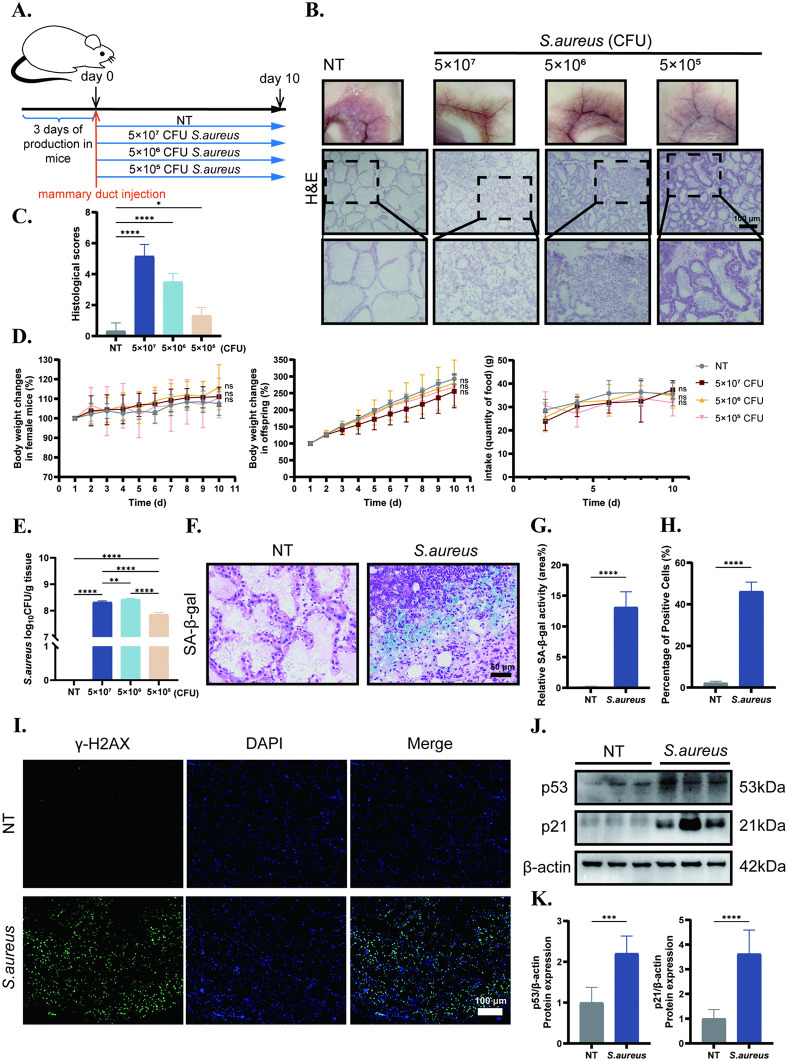
*S. aureus* induces senescence of mouse mammary gland tissue. **A.** Schematic diagram of mouse mammary gland modeling via *S. aureus* stimulation; **B.** Gross pathological changes and H&E staining results of mouse mammary gland tissue; **C.** Histological score of mouse mammary gland tissue; **D.** Changes in body weight of maternal mice, average body weight of pups per litter, and changes in food intake of maternal mice; **E.** Detection results of bacterial load in mammary gland tissue; **F.** SA-β-gal staining results of mammary gland tissue; **G.** Proportion of SA-β-gal-positive area in the staining; **H.** Proportion of positive cells in the immunofluorescence staining; **I.** γ-H2AX immunofluorescence staining results of mammary gland tissue; **J.** Representative Western blot analysis of p53 and p21 protein in mouse mammary gland tissue, with three representative samples shown per group; **K.** Relative protein levels of p53 and p21 normalized to β-actin; Data are presented as mean ± standard deviation (n=6). *p < 0.05, **p < 0.01, ***p < 0.001, ****p < 0.0001. ns indicates no significant difference.

To further verify whether *S. aureus* exerts the same effect *in vivo*, we conducted animal experiments. On day 3 postpartum, lactating female mice were subjected to intraductal injection of *S. aureus* at inoculation doses of 5 × 10⁷, 5 × 10⁶, or 5 × 10⁵ CFU per mammary gland. Mammary gland tissues were collected from the female mice on the 10th day post-injection for subsequent detection (Fig 2A). Gross observation revealed that the mammary glands infected with *S. aureus* at medium and high doses (5 × 10^6^ and 5 × 10^7^ CFU) exhibited severe atrophy, whereas no obvious gross pathological changes were observed in the mammary glands of mice stimulated with *S. aureus* at the low dose (5 × 10^5^ CFU). Hematoxylin and Eosin (H&E) staining results showed that in the high-dose *S. aureus* stimulation group, the acinar walls of mouse mammary glands underwent extensive rupture and atrophy, with a large number of inflammatory cells infiltrating the acinar lumens and interstitial tissues, indicating a strong inflammatory response in the tissues. As the inoculation dose decreased, tissue damage and inflammatory responses gradually diminished; in the low-dose group, the acinar walls thickened, but the inflammatory response was not obvious (Fig 2B and 2C). Fig 2D shows the changes in the body weight of female mice, the average body weight of each litter of pups, and the food intake of female mice from day 1 (d1) to day 10 (d10) post-injection, indicating that intraductal injection of *S. aureus* did not cause a severe systemic response in the mice. Bacterial load analysis of mammary gland tissues showed that the bacterial load in the 5 × 10⁶ CFU group was significantly higher than that in the 5 × 10⁵ and 5 × 10⁷ CFU groups (Fig 2E). Therefore, 5 × 10⁶ CFU was selected for subsequent experiments. SA-β-gal staining showed that *S. aureus* infection increased SA-β-gal-positive staining in mammary gland tissues (Fig 2F and 2G). Similarly, immunofluorescence results also showed that *S. aureus* stimulation led to a significant accumulation of γ-H2AX in mouse mammary gland tissues (Fig 2H and 2I). Additionally, Western Blot results of tissues indicated a significant upregulation in the expression levels of p53 and p21 (Fig 2J and 2K). These results collectively confirm that *S. aureus* stimulation induces senescence in mouse mammary gland tissues.

### Transcriptomic and metabolomic analyses of *S. aureus*-infected mouse mammary tissues

**Fig 3:**
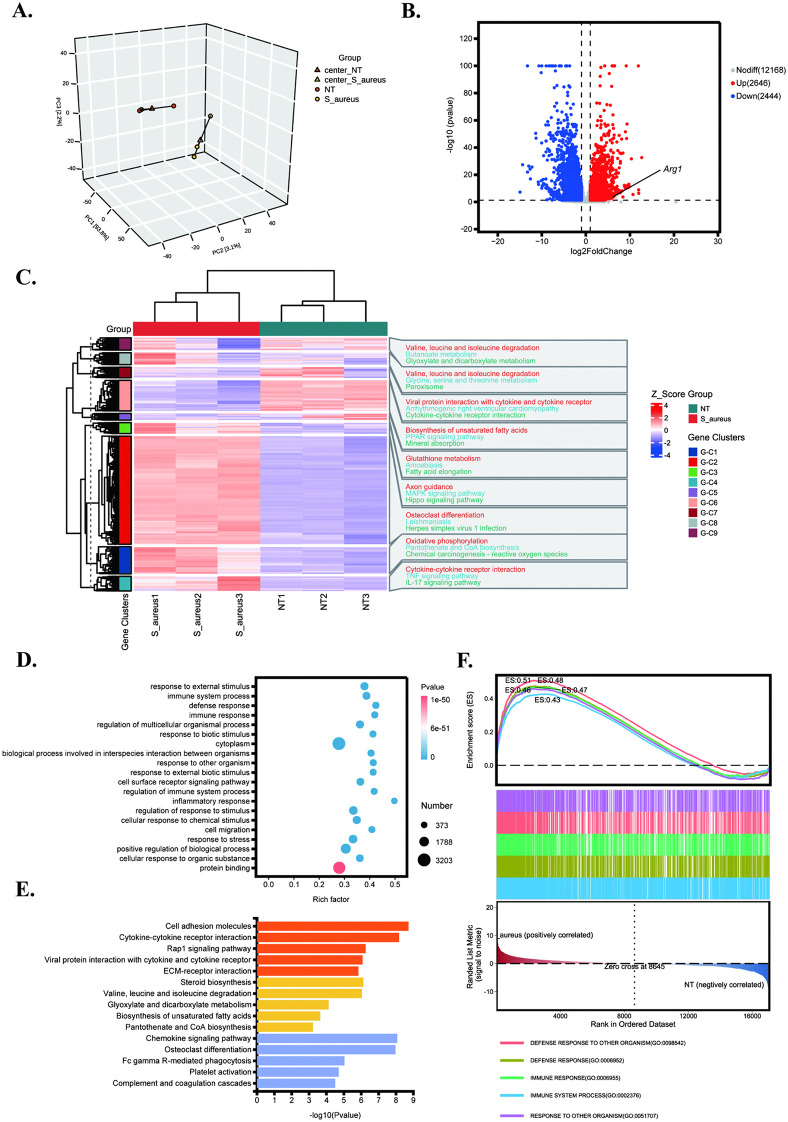
Transcriptome sequencing analysis of mouse mammary gland tissue. **A.** Principal component analysis (PCA) between groups; **B.** Volcano plot analysis between groups; **C.** Heatmap of differentially expressed genes (DEGs) and their enriched pathways; **D.** Gene Ontology (GO) analysis of DEGs; **E.** Kyoto Encyclopedia of Genes and Genomes (KEGG) enrichment analysis of DEGs; **F.** Gene set enrichment analysis (GSEA) of DEGs. This experiment was performed with 3 biological replicates (n = 3).

**Fig 4:**
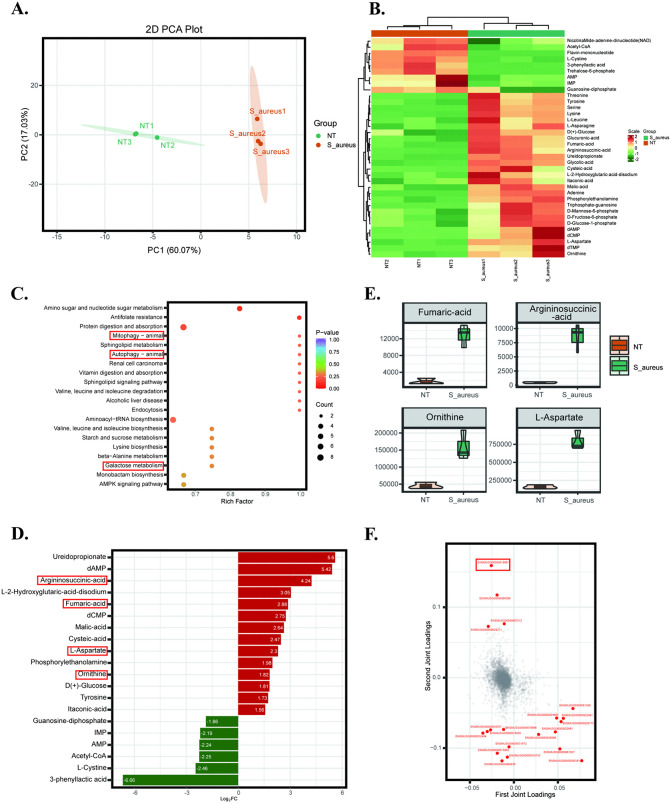
Targeted metabolomics of energy metabolism and multi-omics integration analysis of mouse mammary gland tissue. **A.** Principal component analysis (PCA) between groups; **B.** Heatmap of differential metabolites; **C.** Kyoto Encyclopedia of Genes and Genomes (KEGG) enrichment analysis of differential metabolites; **D.** Top 20 differential metabolites ranked by fold change/variation; **E.** Contents of fumaric acid, argininosuccinic acid, ornithine, and aspartic acid in tissues; **F.** Top 20 genes among transcriptomic differentially expressed genes (DEGs) with the strongest regulatory effects on the metabolome. This experiment was performed with 3 biological replicates (n = 3).

To further explore the mechanism by which *S. aureus* induces mammary gland senescence in mice, we performed transcriptomic sequencing and metabolomic sequencing on mammary gland tissues from healthy mice and *S. aureus*-stimulated mice. First, transcriptome sequencing was performed. Principal component analysis (PCA) and volcano plots (Fig 3A and 3B) revealed extensive transcriptional reprogramming induced by infection, with 2,646 genes significantly upregulated and 2,444 genes significantly downregulated. Fig 3C presents the heatmap of differentially expressed genes and enriched terms between infected and uninfected tissues. MAPK signaling was one of the pathways enriched in infected tissues. GO and KEGG enrichment analyses showed that the differentially expressed genes were mainly associated with immune response, inflammatory response, and response to external biotic stimuli (Fig 3D and 3E). Gene set enrichment analysis (GSEA) revealed substantial enrichment of differentially expressed genes in immune defense–related pathways (Fig 3F). Mitochondrial alterations and associated energetic metabolism exert profound impacts on cellular senescence. To define the functional consequences of these transcriptional changes, we conducted targeted liquid chromatography–tandem mass spectrometry (LC‑MS/MS) metabolomic profiling focused on energy metabolism. Results of PCA revealed a significant difference in energy metabolism between the control group and the *S. aureus* group (Fig 4A). The heatmap exhibited 36 differential metabolites (Fig 4B). KEGG enrichment analysis showed that these differential metabolites were significantly enriched in the autophagy, mitophagy and galactose metabolism pathways (Fig 4C). Metabolomics revealed specific accumulation of urea cycle intermediates. In contrast to the widespread transcriptomic alterations, the metabolomic signature exhibited high specificity. The most markedly elevated metabolites in infected tissues converged on the urea cycle and arginine catabolic pathways (Fig 4D). Specifically, synchronous accumulation was observed for argininosuccinic-acid (log_2_FC = 4.24), fumaric-acid (log_2_FC = 2.88), L‑aspartate (log_2_FC = 2.30), and ornithine (log_2_FC = 1.82) (Fig 4D and 4E). These metabolites are upregulated when mammary tissues are infected with *S. aureus*, indicating a strong activation of the ornithine cycle. To pinpoint the driver genes responsible for this metabolic phenotype, we integrated transcriptomic and metabolomic datasets, focusing on rate‑limiting enzymes of the urea cycle. Notably, Arg1 was identified among the most significantly upregulated genes in the transcriptome (Fig 3B), which perfectly matched the accumulation of its direct enzymatic product, ornithine. To statistically validate this association, we performed bidirectional orthogonal partial least squares (O2PLS) analysis (Fig 4F). Arg1 (ENSMUSG00000019987) exhibited the highest joint loading score, indicating that it serves as the strongest statistical predictor of global metabolic variation. Collectively, these multi‑omics data demonstrate that Arg1 acts as a key metabolic driver that channels arginine into the urea cycle and potentiates ornithine production during *S. aureus* infection in epithelial tissues.

### *S. aureus* stimulates Arg1 upregulation in mouse mammary gland tissues, while Arg1 expression decreases in mMECs *in vitro*

**Fig 5:**
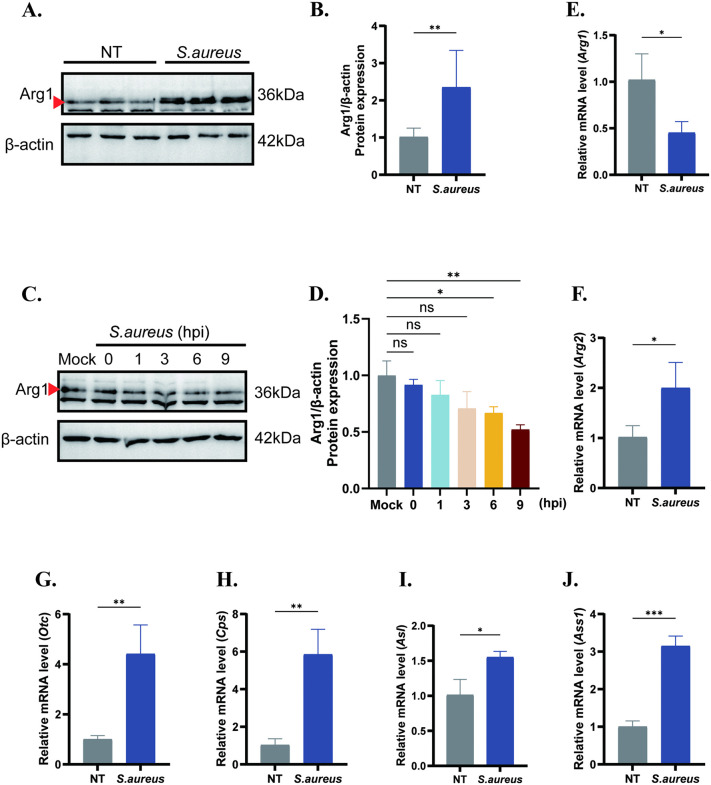
*S. aureus* upregulates Arg1 expression in mouse mammary gland tissue, with different changes in cells. **A.** Representative Western blot analysis of Arg1 protein in mouse mammary gland tissue, with three representative samples shown per group; **B.** Relative protein level of Arg1 normalized to β-actin (in mammary gland tissue) (n = 6); **C.** Western blot analysis of Arg1 protein in mouse mammary epithelial cells (mMECs) (n = 3); **D.** Relative protein level of Arg1 normalized to β-actin (mMECs) (n = 3); **E-J.** mRNA expression levels of *Arg1*, *Arg2*, *Otc*, *Cps*, *Asl*, and *Ass1* in mMECs (n = 3); Data are presented as mean ± standard deviation. *p < 0.05, **p < 0.01, ***p < 0.001. ns indicates no significant difference.

To validate the multi-omics results, we detected the expression level of Arg1 in tissues and cells. Western Blot results showed that *in vivo*, *S. aureus* stimulation induced a significant upregulation of Arg1 expression in mouse mammary gland tissues (Fig 5A and 5B). However, *in vitro*, experiments, the protein expression level of Arg1 in *S. aureus*-stimulated mMECs was decreased (Fig 5C and 5D). To further confirm the expression of Arg1 and the status of ornithine metabolism in cells, we detected the levels of key enzymes involved in the ornithine cycle. qRT-PCR results revealed that *S. aureus* stimulation led to a downregulation of *Arg1* mRNA level in mMECs(Fig 5E). In contrast, the mRNA levels of other enzymes in the cycle, including *Arg2* (*arginase 2*), *Otc* (*ornithine transcarbamylase*), *Cps* (*carbamoyl phosphate synthase*), *Asl* (*argininosuccinate lyase*), and *Ass1* (*argininosuccinate synthase 1*)-were significantly upregulated (Fig 5F–5J). Collectively, these results indicate that *in vivo*, *S. aureus* induces the upregulation of Arg1 expression in mouse mammary gland tissues. However, *in vitro*, *S. aureus* cannot directly induce Arg1 upregulation in mMECs, whereas the mRNA levels of other enzymes in the ornithine cycle are compensatorily upregulated. Therefore, the elevated Arg1 protein in mammary tissues probably derives from macrophage secretion.

### *S. aureus*-stimulated epithelial cells induce Arg1 expression and secretion in macrophages, and macrophage-derived Arg1 may promote epithelial cell senescence *in vitro*

**Fig 6:**
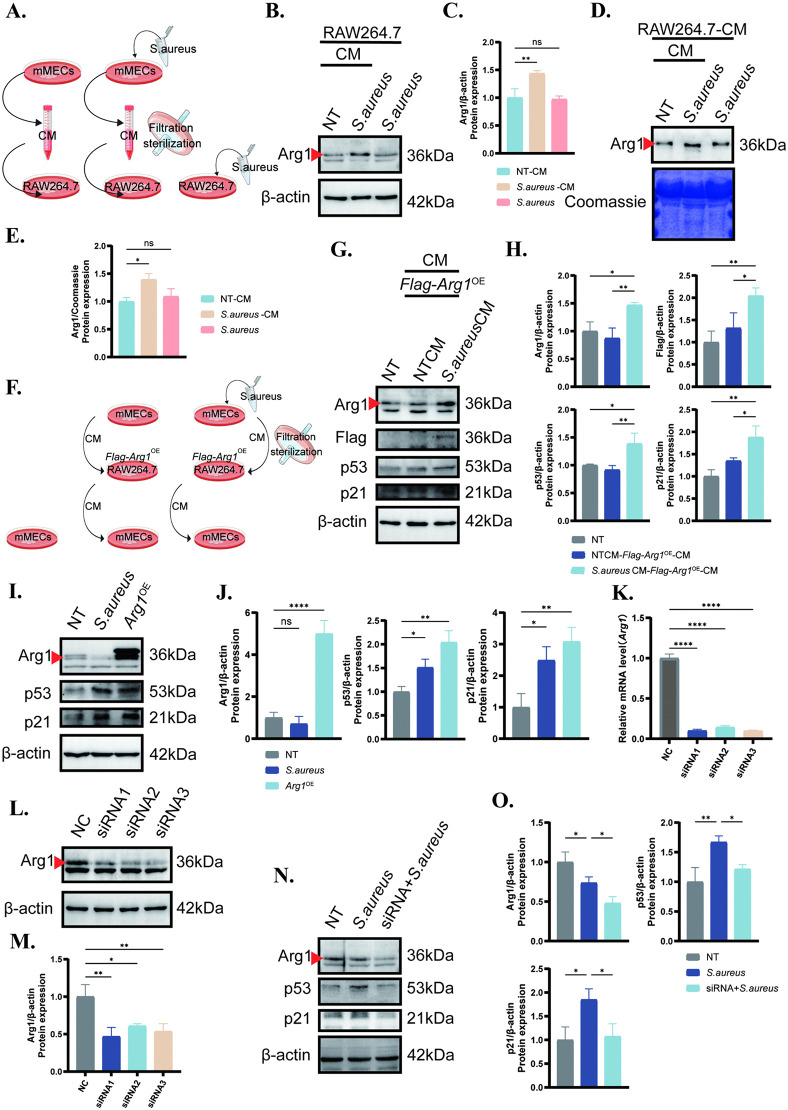
Arg1 derived from macrophages is taken up by mouse mammary epithelial cells (mMECs) and participates in the senescence process. **A.** Schematic diagram of macrophage culture using conditioned medium; **B.** Western blot analysis of Arg1 protein in macrophages; **C.** Relative protein level of Arg1 normalized to β-actin (in macrophages); **D.** Western blot analysis of Arg1 protein in macrophage culture medium; **E.** Relative protein level of Arg1 normalized to Coomassie brilliant blue (in macrophage culture medium); **F.** Schematic diagram of mMEC culture using conditioned medium; **G.** Western blot analysis of Arg1, Flag, p53, and p21 proteins in mMECs; **H.** Relative protein levels of Arg1, Flag, p53, and p21 normalized to β-actin (in mMECs); **I.** Western blot analysis of Arg1, p53, and p21 proteins in mMECs; **J.** Relative protein levels of Arg1, p53, and p21 normalized to β-actin (in mMECs); **K.** mRNA expression level of *Arg1* in mMECs; **L.** Western blot analysis of Arg1 protein in mMECs; **M.** Relative protein level of Arg1 normalized to β-actin (in mMECs); **N.** Western blot analysis of Arg1, p53, and p21 proteins in mMECs; **O.** Relative protein levels of Arg1, p53, and p21 normalized to β-actin (in mMECs); Data are presented as mean ± standard deviation (n = 3). *p < 0.05, **p < 0.01, ****p < 0.0001. ns indicates no significant difference.

Because the expression pattern of Arg1 observed in mammary tissues was not fully consistent with that in epithelial cells cultured alone, we employed a conditioned-medium co-culture model to explore a potential cellular source of Arg1 under *S. aureus* stimulation. Conditioned media were first collected from mMECs with or without *S. aureus* stimulation, sterilized by filtration, and then used to culture RAW264.7 cells (Fig 6A). Western blot analysis showed that conditioned medium from *S. aureus*-stimulated epithelial cells significantly increased Arg1 expression in macrophages (Fig 6B and 6C). In addition, Arg1 content in macrophage-conditioned medium was also significantly elevated (Fig 6D and 6E). *In vitro* CCK-8 and LDH assays confirmed the absence of macrophage death (S1 Fig). These findings suggest that soluble factors released by *S. aureus*-stimulated epithelial cells can induce Arg1 expression in macrophages and promote its release into the extracellular environment in vitro.

To further investigate whether macrophage-derived Arg1 affects epithelial cells, RAW264.7 cells were transfected with a Flag-Arg1 overexpression plasmid. These cells were then cultured with conditioned media from epithelial cells with or without S. aureus stimulation, after which macrophage-conditioned media were collected and used to culture epithelial cells (Fig 6F). Western blot analysis of epithelial cells showed that Arg1, Flag, p53, and p21 protein levels were significantly increased (Fig 6G and 6H). Together, these results suggest that, in this in vitro conditioned-medium model, macrophage-derived Arg1 may be transferred to epithelial cells and may contribute to the upregulation of senescence-associated proteins. However, these data do not definitively establish macrophages as the major source of Arg1 in vivo, and further in situ or in vivo validation is required.

To further assess the role of Arg1 in epithelial cell senescence, we overexpressed Arg1 directly in epithelial cells. Western blot results showed that both *S. aureus* stimulation and Arg1 overexpression significantly increased p53 and p21 protein levels in epithelial cells (Fig 6I and 6J), consistent with the induction of a senescence-associated phenotype. We next knocked down Arg1 in epithelial cells using siRNA. qRT-PCR and Western blot analyses confirmed that both the mRNA and protein levels of Arg1 were significantly reduced after knockdown (Fig 6K–6M). When Arg1-knockdown epithelial cells were stimulated with *S. aureus*, the induction of p53 and p21 was markedly attenuated (Fig 6N and 6O). These findings suggest that Arg1 contributes to the upregulation of senescence-associated proteins in *S. aureus*-stimulated mMECs and may be required for this response under the present *in vitro* experimental conditions.

### The arginine/ornithine cycle drives senescence in mMECs and mouse mammary tissues under excessive substrate stimulation

**Fig 7:**
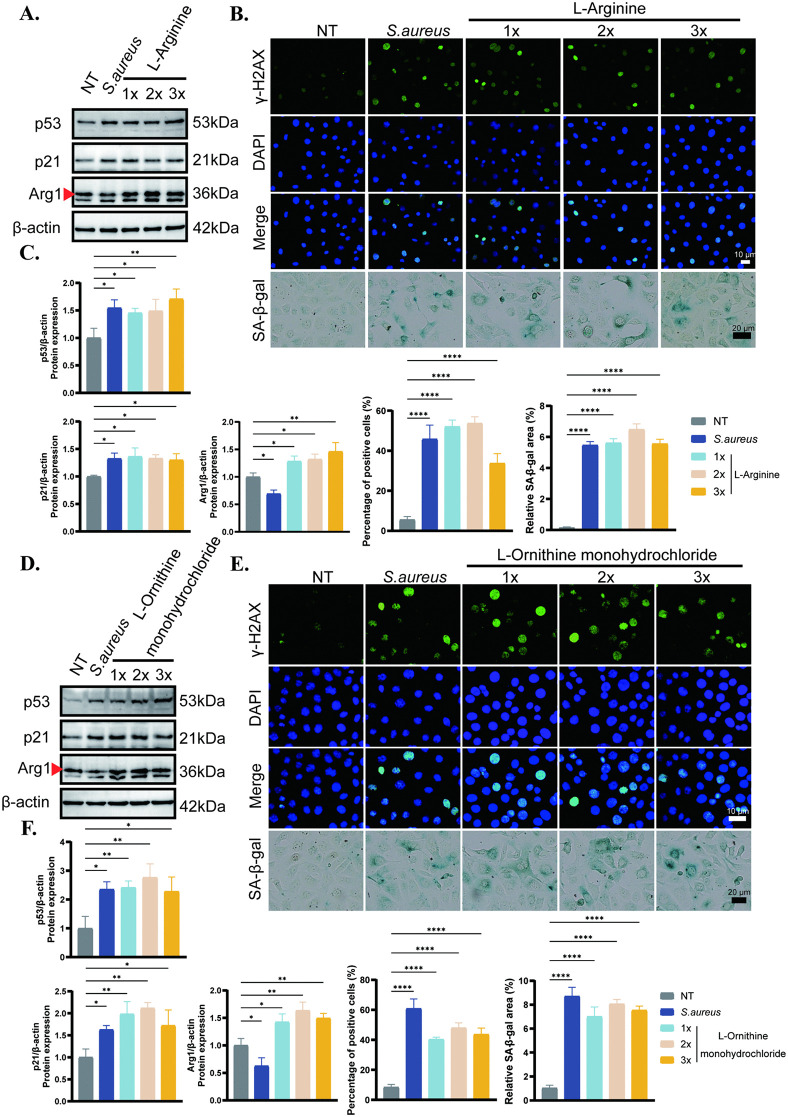
Excess arginine and ornithine hydrochloride induce senescence in mMECs. **A.** Western blot analysis of p53, p21, and Arg1 proteins in mMECs; **B.** γ-H2AX immunofluorescence staining and SA-β-gal staining results of mMECs; **C.** Relative protein levels of p53, p21, and Arg1 normalized to β-actin, proportion of positive cells in immunofluorescence staining, and proportion of positive area in SA-β-gal staining (of mMECs); **D.** Western blot analysis of p53, p21, and Arg1 proteins in mMECs; **E.** γ-H2AX immunofluorescence staining and SA-β-gal staining results of mMECs; **F.** Relative protein levels of p53, p21, and Arg1 normalized to β-actin, proportion of positive cells in immunofluorescence staining, and proportion of positive area in SA-β-gal staining (of mMECs); Data are presented as mean ± standard deviation (n = 3). *p < 0.05, **p < 0.01, ***p < 0.001, ****p < 0.0001. ns indicates no significant difference.

To explore the mechanism by which Arg1 mediates *S. aureus*-induced senescence in mMECs, we further supplemented excess arginine-which promotes Arg1 expression via substrate activation-and conducted *in vitro* experiments using arginine and its metabolite ornithine. Using the ornithine concentration and fold upregulation obtained by metabolomics as references, excess L-arginine or L-ornithine hydrochloride was added to mMECs for induction at the reference dose (1×), with higher doses (2× and 3×) used for validation. Western blot results showed that arginine stimulation significantly upregulated the protein expression levels of Arg1, p53, and p21 in mMECs (Fig 7A and 7C). Immunofluorescence staining showed that arginine treatment increased the nuclear γ-H2AX signal in cells (Fig 7B and 7C). SA-β-gal staining results revealed that arginine stimulation induced a significant accumulation of SA-β-gal in cells (Fig 7B and 7C). Similarly, stimulation of mMECs with ornithine hydrochloride upregulated the protein expression levels of Arg1, p53, and p21 in cells, and led to a significant accumulation of γ-H2AX and SA-β-gal in cells (Fig 7D–7F). Collectively, these results indicate that increasing the levels of arginine and ornithine can further upregulate Arg1 expression and induce senescence in mMECs.

**Fig 8:**
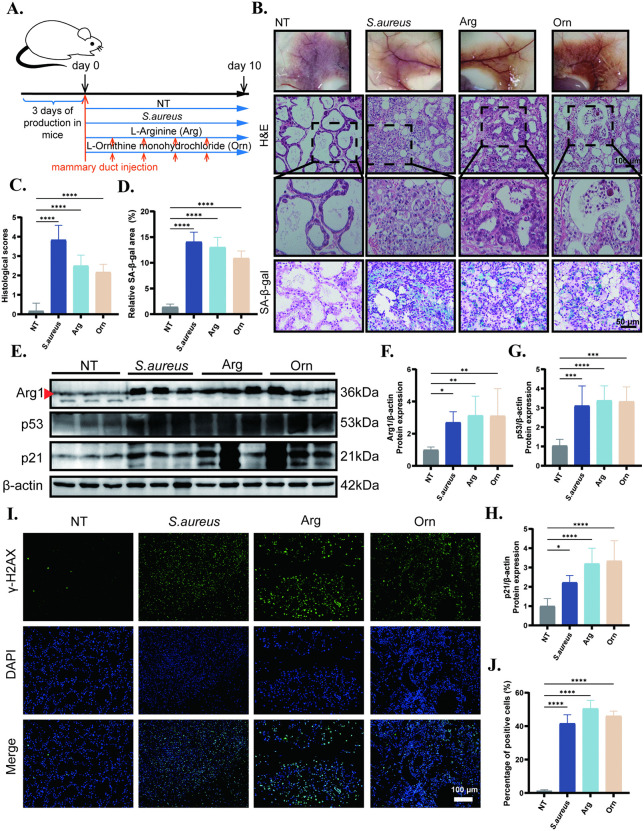
Excess arginine and ornithine induce senescence of mouse mammary gland tissue. **A.** Schematic diagram of mouse mammary gland modeling via arginine and ornithine stimulation; **B.** Gross pathological changes, H&E staining, and SA-β-gal staining results of mouse mammary gland tissue; **C.** Histological score of mouse mammary gland tissue; **D.** Proportion of SA-β-gal-positive area in the staining; **E.** Representative Western blot analysis of Arg1, p53 and p21 protein in mouse mammary gland tissue, with three representative samples shown per group; **F-H.** Relative protein levels of Arg1, p53, and p21 normalized to β-actin (in mammary gland tissue); **I.** γ-H2AX immunofluorescence staining results of mammary gland tissue; **J.** Proportion of positive cells in the immunofluorescence staining; Data are presented as mean ± standard deviation (n = 6). *p < 0.05, **p < 0.01, ***p < 0.001, ****p < 0.0001. ns indicates no significant difference.

We also conducted *in vivo* experiments to verify the effects of arginine and ornithine on mouse mammary glands. Based on the results obtained from metabolomic analyses, excess L-arginine and L-ornithine hydrochloride were administered to mice via intraductal injection of the mammary gland (Fig 8A). H&E staining results showed that intraductal injection of *S. aureus* severely disrupted the integrity of mammary acini in mice, with a large number of inflammatory cells infiltrating the tissues (Fig 8B). In contrast, in mammary gland tissues injected with arginine or ornithine, the acinar walls were significantly thickened, and only a small number of inflammatory cells were observed in the tissues (Fig 8B and 8C). SA-β-gal staining results also demonstrated that injection of arginine or ornithine increased SA-β-gal-positive staining in mammary gland tissues (Fig 8B and 8D). Western blot results revealed that both arginine and ornithine significantly upregulated the protein expression levels of Arg1, p53, and p21 in the tissues (Fig 8E–8H). Additionally, immunofluorescence staining showed that arginine and ornithine increased γ-H2AX signal in mouse mammary gland tissues (Fig 8I and 8J). Collectively, these results indicate that *in vivo*, excess arginine and ornithine can significantly promote the protein expression of Arg1 and induce senescence in mammary gland tissues.

## Discussion

By combining *in vitro* and *in vivo* experiments with multi-omics analysis approaches, this study confirmed that *S. aureus* drives mammary epithelial cell senescence by activating the arginine/ornithine pathway via Arg1-dependent macrophage-epithelial cell crosstalk. These findings provide experimental evidence for elucidating the mechanisms of chronic infection-associated tissue senescence and developing targeted interventional strategies.

*S. aureus*‑induced senescence in mammary epithelial cells represents a multifaceted process involving DNA damage, activation of the SASP [32]. This study confirmed in vitro that *S. aureus* stimulation upregulated senescence markers such as p53 and p21, enhanced γ‑H2AX fluorescence intensity, and promoted the secretion of SASP‑associated cytokines including IL‑1β and IL‑6. These results are consistent with the general pathological mechanism reported in previous studies, whereby pathogens induce cellular senescence through various pathways such as DNA damage [33–37]. This suggests that pathogen-induced senescence in target cells may represent a universally existing pathological mechanism.

A core scientific question addressed in this study concerns the cellular origin of aberrantly upregulated Arg1 in mammary tissue following *S. aureus* infection. Multi‑omics analyses identified Arg1 as a central regulatory gene of the ornithine cycle, and *in vivo* experiments confirmed elevated Arg1 expression in mammary tissue. However, mammary epithelial cells cultured in vitro did not exhibit Arg1 upregulation upon *S. aureus* stimulation; instead, compensatory upregulation of other enzymes was observed in the ornithine cycle. This discrepancy prompted us to investigate non‑epithelial sources of Arg1 within the mammary microenvironment. Previously, Arg1 was considered a cytosolic enzyme localized within cells [26]. In this study, upon *S. aureus* stimulation, cytokines secreted by mammary epithelial cells induced robust expression and secretion of Arg1 by macrophages into the microenvironment. Subsequently, this secreted Arg1 was internalized by mammary epithelial cells, driving the conversion of arginine to ornithine. These findings are consistent with previous reports that Arg1 can be secreted by immune cells via EVs to mediate intercellular communication [27,28]. Notably, high expression of Arg1 in macrophages is not only a key step in the interaction between macrophages and epithelial cells but also reflects phenotypic transformation of macrophages in the chronic inflammatory microenvironment. Abundant expression of Arg1 confers anti-inflammatory M2 phenotypes on macrophages, which is intended to suppress excessive inflammatory reactions and reduce tissue damage [38]. However, in this study, this adaptive response unexpectedly triggered metabolic dysfunction in epithelial cells and promoted tissue senescence, revealing the dual role of macrophage phenotypic switching during chronic infection.

There were significant differences in senescent phenotypes between in vivo and in vitro experiments in this study, which represents an important feature of the present work. The upregulation level of senescence markers in mammary tissues was significantly higher than that in *vitro* cultured epithelial cells. The important reason is that the in vivo mammary microenvironment constitutes a complex network involving multiple cell types and signaling pathways. This study demonstrates that direct crosstalk between macrophages and epithelial cells mediated by Arg1 represents a novel mechanism driving senescence. *In vivo,* abundant Arg1 secreted by macrophages promotes the ornithine cycle in epithelial cells and also leads to substantial local depletion of arginine in the microenvironment. Arginine deficiency, in turn, suppresses the proliferation and activation of immune cells, thereby remodeling the immune microenvironment [39]. This not only facilitates persistent colonization of *S. aureus* and maintenance of chronic infection but also chronic infection-induced abundant inflammatory factors, such as TGF-β, further induce senescence in adjacent cells [40]. Moreover, reduced nitric oxide (NO) production resulting from substrate competition for arginine [41] further impairs host immune defense and oxidative stress resistance. Collectively, these factors synergistically exacerbate mammary tissue senescence. These findings also suggest that *S. aureus*‑mediated regulation of mammary aging is a multi‑factor synergistic process, and single‑cell in vitro models can only partially recapitulate the underlying molecular mechanisms.

This study presents several important innovations in the field of chronic infection and tissue senescence. First, it identifies the Arg1-ornithine axis as a novel regulatory pathway driving mammary senescence induced by chronic *S. aureus* infection. Second, it reveals an intercellular communication pattern between macrophages and mammary epithelial cells mediated by extracellular Arg1, expanding our understanding of cell crosstalk within the mammary microenvironment under chronic inflammation. Third, it establishes a link between the ornithine cycle and mammary senescence, providing a new direction for investigating metabolic disorders in mammary diseases.

Notably, this study also has certain limitations that require further refinement in subsequent research. The *in vivo* model used in this study is a short-term chronic mastitis model established by injecting *S. aureus* into the mammary ducts of lactating mice. This study confirms that macrophages are one of the cellular sources of Arg1; however, the specific cytokines secreted by mammary epithelial cells that induce Arg1 expression in macrophages remain undefined, and the downstream signaling pathways underlying Arg1 upregulation in macrophages warrant in-depth exploration. Additionally, the mammary microenvironment consists of multiple cell types. This study only investigated the interaction between macrophages and mammary epithelial cells and merely verified macrophages as one of the Arg1 sources. It should be noted that the conclusion regarding macrophages as a source of Arg1 is currently based on an in vitro conditioned-medium model. Because *in vitro* cellular responses may not fully recapitulate the *in vivo* microenvironment of *S. aureus*-infected mammary tissue, further in situ localization and *in vivo* validation are needed to determine the relative contribution of macrophages and epithelial cells to Arg1 production.

What requires key discussion is the relevance and limitations of the animal model used in this study. This study established a chronic mastitis model induced by *S. aureus* using lactating ICR mice. Although this model recapitulates the local inflammatory characteristics and tissue dysfunction phenotypes of clinical chronic mastitis, it should be clarified that mice are not the natural host of *S. aureus*. Under natural conditions, *S. aureus* preferentially colonizes the mammary tissues of humans and ruminants such as cattle and sheep, leading to mastitis [32,42–44]. This host specificity difference may cause certain discrepancies between the experimental model and natural infection scenarios [45], potentially affecting Arg1 secretion efficiency and the intensity of immune metabolic regulation. Studies have shown that Arg1-mediated metabolic pathways are evolutionarily conserved [23], providing mechanistic insights and potential targets for human mastitis or other animal infection models. However, further validation using bovine mammary epithelial cell models or humanized mouse models in future studies is required to confirm the applicability of this mechanism in natural hosts.

Under chronic infection, aberrant crosstalk between macrophages and epithelial cells disrupts metabolic pathway homeostasis, driving functional senescence of mammary tissue. The clinical implication of this finding is that therapeutic strategies can be developed to target Arg1 or ornithine metabolism. Meanwhile, this study suggests that metabolites such as Arg1 and ornithine could serve as potential biomarkers for chronic mastitis. Detection of these molecules in patient milk or serum may enable early prediction of tissue senescence risk, thereby providing an optimal window for clinical intervention.

In summary, this study systematically elucidates the molecular mechanism by which *S. aureus* drives mammary tissue aging through macrophage-epithelial crosstalk mediated by the Arg1-ornithine cycle. Despite limitations regarding host specificity of the animal model, the immune–metabolic regulatory axis uncovered here exhibits potential cross‑species conservation, providing a new paradigm for understanding common mechanisms underlying chronic infection‑associated tissue senescence. These findings not only refine the theoretical framework of chronic infection‑induced tissue senescence but also identify novel potential therapeutic targets for clinical management of persistent *S. aureus* mastitis. Targeting the Arg1-ornithine axis to suppress aberrant activation of the ornithine cycle may represent a promising strategy to delay mammary aging and improve secretory function in patients with chronic mastitis. Furthermore, this study offers new perspectives for investigating other chronic infection‑related senescent disorders and provides important mechanistic references for developing metabolic interventions against tissue aging.

## Materials and methods

### Ethics statement

This study was approved by the Experimental Animal Welfare and Ethics Committee of Jilin University. All animal experiments were conducted in strict compliance with the guidelines of the institutional ethics committee (Protocol No.:SY202508026).

### Cell culture

The mouse mammary epithelial cell line EpH4-Ev and the mouse peritoneal macrophage cell line RAW264.7 were both purchased from the American Type Culture Collection. All cells were cultured in high-glucose DMEM medium (Solarbio, China) supplemented with 10% fetal bovine serum (Ausail, China) in a 37°C incubator with 5% CO_2_. For mouse mammary epithelial cells (mMECs), passage was performed when the cell confluency reached 80%. When the cell confluency reached approximately 90%, *S. aureus* was added for stimulation for 1.5 hours (multiplicity of infection, MOI = 10). The medium was then discarded, and the cells were washed three times with phosphate-buffered saline (PBS). Fresh medium containing gentamicin at a final concentration of 100 μg/mL was added for 1-hour culture, and this time point was recorded as 0 hours post-infection (hpi). Finally, the cells were washed three times with PBS and cultured in fresh medium.

Alternatively, the cells were stimulated with arginine (Aladdin, China) (300 μM (1×), 600 μM (2×), and 900 μM (3×)) or ornithine hydrochloride (Aladdin, China) (765 μM (1×), 1530 μM (2×) and 2295 μM (3×)) for 9 hours. For RAW264.7 cells, passage was conducted when the cell confluency reached 80%. For indirect co-culture of cells: conditioned medium was used for indirect co-culture. The obtained conditioned medium was centrifuged to remove cell debris, followed by filtration for sterilization, and then used to culture other cells.

### Bacterial culture

The *S. aureus* strain was purchased from the American Type Culture Collection (ATCC 35556). The strain was cultured in LB broth medium (Tryptone 2 g, Yeast extract 1 g, NaCl 2 g, Deionized water 200 mL) at 37°C for 12 hours. The bacterial culture was then collected, washed and resuspended with phosphate-buffered saline (PBS), and the bacterial count was quantified.

### Western blotting

The collected cells and tissue samples were lysed using NP‑40 lysis buffer supplemented with 1 mM PMSF. After centrifugation at 12,000 rpm, the supernatants were harvested. Protein concentrations were quantified using a BCA assay kit (Thermo Scientific, USA) and normalized to ensure equal loading across all samples. Proteins were separated by 12% SDS‑PAGE and transferred onto PVDF membranes (Millipore, Germany). Membranes were blocked with 5% nonfat milk, incubated with primary antibodies (1:1000 dilution) overnight at 4 °C, and subsequently probed with secondary antibodies (1:5000 dilution). Immunoreactive bands were visualized using enhanced chemiluminescence (ECL) substrate (Yeasen Biotechnology, China) and detected with a chemiluminescence imaging system (Tanon Science & Technology, China).

Antibody information: Primary antibodies against p53, p21, Arg1, Flag, and β‑actin (Proteintech, China). Secondary antibodies including goat anti‑rabbit IgG and goat anti‑mouse IgG (Boster, USA).

### Senescence-associated β-galactosidase (SA-β-gal) staining

Processed cell samples or frozen sections of tissues were stained using an SA-β-gal staining kit (Beyotime, China). After washing the cells with phosphate-buffered saline (PBS), the cells were fixed. Subsequently, the fixed cells were incubated with the staining working solution in a 37°C incubator without CO_2_ for staining. For tissue sections, eosin was used to stain the cell nuclei.

### DNA damage detection

First, routine deparaffinization and rehydration procedures were performed, followed by staining. For staining processed cell samples or paraffin‑embedded tissue sections, a DNA Damage Detection Kit (γ‑H2AX Immunofluorescence) (Beyotime Biotechnology, China) was used. Fixation and blocking were completed using the fixative and blocking buffer provided in the kit. Samples were incubated with the primary and secondary antibodies supplied with the kit and washed using the washing buffer included in the kit. Nuclei were counterstained with DAPI.

### Quantitative real-time PCR (qRT-PCR)

Total RNA was extracted from cell or tissue samples using the TRIzol reagent (Thermo Fisher, USA). RNA quality and quantity were assessed by measuring the A260/280 ratio using a Nanodrop spectrophotometer (ALLSHENG, China). First-strand complementary DNA (cDNA) was synthesized by reverse transcription from the extracted RNA using a cDNA First-Strand Synthesis Kit (ABclonal, China). Subsequently, qRT-PCR was performed with the Quantitect SYBR Green RT-PCR Kit (Roche, China) according to the manufacturer’s instructions on a QuantStudio 1 Real-Time PCR System (Thermo Fisher, USA). Primer sequences, amplicon lengths, and accession numbers of reference genes are listed in Table 1. The melting curves of qPCR products showed a single specific peak, and no obvious amplification was observed in no-template control and no-reverse-transcription control groups. The stability of candidate reference genes including β-actin, GAPDH, and Ppia was evaluated using geNorm software. GAPDH was selected as the optimal reference gene for normalization, meeting the requirements for reliable internal control usage. Relative gene expression levels were calculated using the 2^(−ΔΔCt) method. Three biological replicates were set for each group, and experimental results are presented as mean ± standard deviation.

**Table 1:** Primer sequences.

Gene	Forward primer (5’-3’)	Reverse primer (5’-3’)	Product length (bp)
*IL-1β*	TCGCAGCAGCACATCAACAAGAG	AGGTCCACGGGAAAGACACAGG	97
*IL-6*	TTCTTGGGACTGATGCTGGTGAC	GTGGTATCCTCTGTGAAGTCTCCTC	80
*TNF-a*	GCCTCTTCTCATTCCTGCTTGTGG	GTGGTTTGTGAGTGTGAGGGTCTG	149
*CXCL1*	ATGGCTGGGATTCACCTCAAGAAC	AGTGTGGCTATGACTTCGGTTTGG	89
*CXCL3*	CACTGGTCCTGCTGCTGCTG	CGTCACCGTCAAGCTCTGGATG	134
*CXCL5*	ATCCCCAGCGGTTCCATCTCG	CGTTGCGGCTATGACTGAGGAAG	117
*Arg1*	CTCCAAGCCAAAGTCCTTAGAG	AGGAGCTGTCATTAGGGACATC	185
*Arg2*	AGGAGTGGAATATGGTCCAGC	AGGGATCATCTTGTGGGACATT	125
*Otc*	ACACTGTTTGCCTAGAAAGCC	CCATGACAGCCATGATTGTCC	113
*Cps*	ACATGGTGACCAAGATTCCTCG	TTCCTCAAAGGTGCGACCAAT	119
*Asl*	CTATGACCGGCATCTGTGGAA	AGCAACCTTGTCCAACCCTTG	127
*Ass1*	ACACCTCCTGCATCCTCGT	GCTCACATCCTCAATGAACACCT	149
*Gapdh*	TGTGTCCGTCGTGGATCTGA	TTGCTGTTGAAGTCGCAGGAG	150

### Animals and experimental design

Eight-week-old ICR mice were purchased from Liaoning Changsheng Biotechnology Co., Ltd. After one week of adaptive feeding, two female mice were caged with one male mouse, and all mice had free access to food and water. Upon confirmation of pregnancy, the female mice were transferred to new cages. On day 3 postpartum, lactating female mice were randomly divided into four groups, with six mice per group: a control group and three S. aureus-inoculated groups. Mice in the *S. aureus*-inoculated groups received intraductal injection of 50 μL *S. aureus* suspension into each mammary gland at concentrations of 10⁹, 10⁸, or 10⁷ CFU/mL, corresponding to final inoculation doses of 5 × 10⁷, 5 × 10⁶, or 5 × 10⁵ CFU per mammary gland, respectively. These groups were used to establish and evaluate a mouse model of S. aureus-induced mammary senescence. Two hours before induction, the female mice were separated from their pups. After anesthetizing the mice via intraperitoneal injection of sodium pentobarbital, different doses of *S. aureus* bacterial suspension were injected into the 4th pair of mammary ducts of each mouse, with a volume of 50 μL per mammary gland. After induction, the number of pups per litter was reduced to 5. During the induction period, the body weights of the female mice and pups, as well as the food intake of the female mice, were monitored. On the 10th day post-induction, the mice were euthanized, and their mammary gland tissues were collected. Alternatively, arginine (12mM) or ornithine hydrochloride (30.6mM) was injected into the mammary ducts of the mice (50 μL per mammary gland, once every 2 days). The control group received an injection of the same volume of normal saline. Mammary gland tissue samples were collected on the 10th day after model establishment. For sample allocation, 3 mice per group were randomly selected for transcriptomic and metabolomic analysis, and 6 mice per group were used for phenotypic, histological, biochemical and molecular verification experiments.

### Hematoxylin and Eosin (H&E) staining and histopathological scoring

Mammary tissues fixed in 4% paraformaldehyde were embedded in paraffin and sectioned at 5 μm thickness. These sections were deparaffinized in xylene and rehydrated through a graded ethanol series (100%, 95%, 90%, 80%, and 70%). Subsequently, the sections were stained with hematoxylin solution (Solarbio, China) for 5–10 min, differentiated in 1% hydrochloric acid–alcohol, and rinsed in tap water. The sections were then counterstained with eosin solution (Solarbio, China). Finally, all sections were dehydrated in sequentially increasing ethanol concentrations, cleared in xylene, and mounted with neutral balsam. Images were captured using an optical microscope. The scoring criteria were as follows: 0 = intact mammary acini or no inflammatory cell infiltration; 1 = slight damage to acini or mild inflammatory cell infiltration; 2 = moderate damage to acini or moderate inflammatory cell infiltration; 3 = extensive damage to acini or severe inflammatory cell infiltration.

### Tissue bacterial load detection

Under sterile conditions, mouse mammary tissues were harvested, weighed, and homogenized for 10 min in sterile phosphate-buffered saline (PBS) containing magnetic beads at a 1:9000 tissue weight dilution. Homogenates were serially diluted 1000-fold and 10,000-fold, plated onto LB agar plates, and incubated for 24 h before colony enumeration.

### Transcriptomic and metabolomic sequencing

#### Transcriptomic sequencing.

Total RNA was extracted from mammary gland tissues using TRIzol reagent. The integrity and concentration of RNA were evaluated using an Agilent 2100 Bioanalyzer. cDNA libraries were constructed by Shanghai Personal Biotechnology Co., Ltd. and sequenced on the Illumina NovaSeq 6000 platform. Raw sequencing reads were filtered to remove adapter sequences and low-quality reads. The DESeq2 R package was used to identify differentially expressed genes (DEGs) with the screening thresholds set as |log_2_FoldChange| > 1 and *P < 0.05*.

#### Targeted metabolomic analysis.

Targeted metabolomics analysis focusing on energy metabolism was performed on mouse mammary gland tissues by Shanghai Personal Biotechnology Co., Ltd. In the metabolomics analysis, after pretreatment of tissue samples, metabolites were extracted using a methanol/water mixture. Ultra-high-performance liquid chromatography coupled with tandem mass spectrometry (UHPLC-MS/MS) was employed. Qualitative identification and quantitative analysis of targeted metabolites were completed based on standard retention times and multiple reaction monitoring (MRM) ion pairs. Differential metabolites between groups were screened with the criteria of VIP > 1, fold change ≥ 2.

### CCK-8 assay

The assay was performed using CCK-8 (Invigentech, USA). For the treated cells, the culture medium was replaced with fresh medium containing CCK solution at a ratio of 10:1, followed by incubation for 1–4 hours. The absorbance value was then measured at a wavelength of 450 nm.

### LDH assay

The assay was conducted using an LDH Cytotoxicity Assay Kit (Beyotime, China). The LDH release reagent provided with the kit was added to the cells for 1 hour of pretreatment to serve as the positive control. The cell culture supernatant was collected and centrifuged, after which the prepared LDH assay working solution was added. The mixture was incubated for 30 minutes in the dark, and the absorbance was measured at a wavelength of 490 nm.

### RNA interference and overexpression

Plasmid construction: To construct the Arg1 overexpression plasmid, the full-length coding sequence of mouse Arg1 (GenBank accession number: NM_007482) was amplified and cloned into the pcDNA3.1 vector (Runze Biological, China). A 3 × Flag tag was fused to the C-terminus of the Arg1 protein to generate the overexpression plasmid (Flag-Arg1^OE^). The empty vector was used as the negative control (NT).

siRNA knockdown plasmids: For RNA interference, small interfering RNA (siRNA) plasmids targeting Arg1 (siRNA1, siRNA2, and siRNA3) and a non-targeted scrambled control plasmid (NC) were constructed (Runze Biological, China). Detailed sequences are listed in S1 Table.

Transfection protocol: Cells were seeded in 6 cm culture dishes and cultured to approximately 40% confluence. Transfection was performed using Lipofectamine 2000 (Thermo Fisher Scientific, USA) following the manufacturer’s protocol for plasmid DNA transfection. Each dish was transfected with 5 µg overexpression plasmid or 5 µg shRNA plasmid. The medium was replaced with fresh complete medium 6 hours after transfection. Cells were harvested 24 hours post-transfection. The efficiency of overexpression and knockdown was validated by Western blotting.

### Data analysis

Data analysis was performed using GraphPad Prism 8 software. For comparisons between two groups, an independent samples t-test was used. Each value is presented as the mean ± standard deviation (SD), and a difference was considered statistically significant when *p < 0.05*. For comparisons among multiple groups, one-way analysis of variance (ANOVA) was used. Each value is presented as the mean ± standard deviation (SD), and a difference was considered statistically significant when *p < 0.05*.

## Supporting information

S1 Table Plasmid sequence. (XLSX)

S1 Fig Viability of macrophages after stimulation with epithelial cell culture medium. A. Macrophages were cultured with conditioned medium from mMECs either unstimulated or stimulated with *S. aureus*, and macrophage viability was detected using CCK-8 assay. B. Macrophages were cultured with conditioned medium from mMECs either unstimulated or stimulated with *Staphylococcus aureus*, and LDH release by macrophages was measured (with LDH release agent used as a positive control). Data are presented as mean ± standard deviation. ****p < 0.0001. ns indicates no significant difference.
(TIF)
