# Supplementary figures and images for: Staphylococcus aureus rewires arginine metabolism to drive mammary aging via macrophage–epithelial crosstalk
Source: PLoS Pathog. 2026 Jul 6;22(7):e1014403. doi: 10.1371/journal.ppat.1014403 (PMC13349301; doi:10.1371/journal.ppat.1014403)

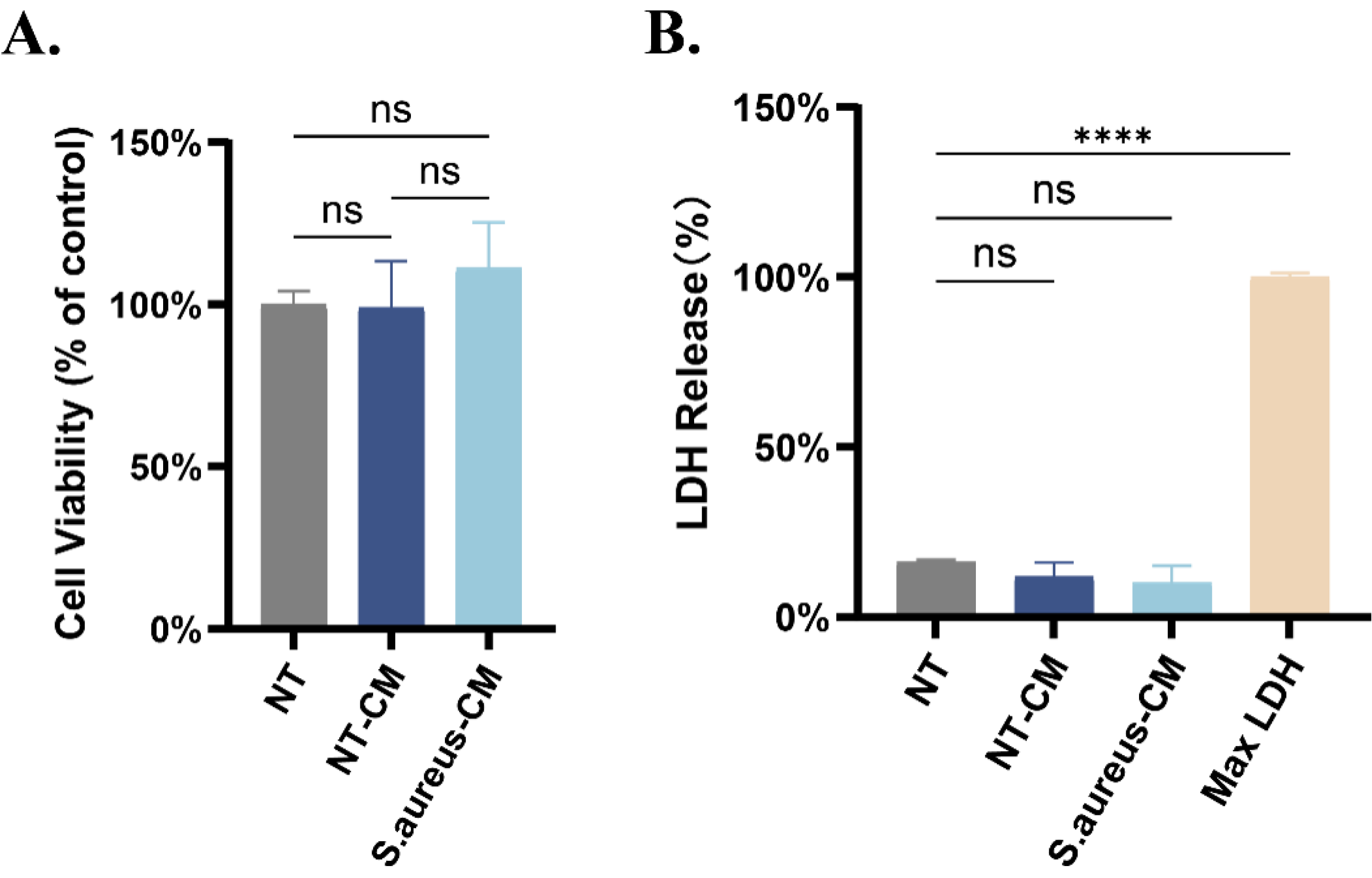

Supplement: S1 Fig — A. Macrophages were cultured with conditioned medium from mMECs either unstimulated or stimulated with S. aureus, and macrophage viability was detected using CCK-8 assay. B. Macrophages were cultured with conditioned medium from mMECs either unstimulated or stimulated with Staphylococcus aureus, and LDH release by macrophages was measured (with LDH release agent used as a positive control). Data are presented as mean ± standard deviation. ****p < 0.0001. ns indicates no significant difference. (TIF) [file ppat.1014403.s002.tif]
